# Male circumcision uptake and its predictors among sexually active men aged 15–59 years living in the highest HIV prevalence region of Ethiopia: evidence from 2016 Ethiopia demographic and health survey

**DOI:** 10.1186/s12889-020-09918-5

**Published:** 2020-11-23

**Authors:** Zerihun Kura Edossa, Abonesh Taye Kumsa, Mamo Nigatu Gebre

**Affiliations:** 1 Department of Epidemiology, Jimma University, Institute of Health, Faculty of Public Health, Jimma, Ethiopia; 2 Department of Nutrition and Dietetics, Jimma University, Institute of Health, Faculty of Public Health, Jimma, Ethiopia

**Keywords:** Male circumcision, Sexually active men, 2016 EDHS

## Abstract

**Background:**

Pieces of evidence showed that the Gambella region of Ethiopia has remained HIV hotspot area for successive years. However, the magnitude of male circumcision uptake and its associated factors are not well studied in this region. Hence, the aim of the current study is to assess the magnitude of male circumcision uptake and its predictors among sexually active men in the region using the 2016 Ethiopian Demographic and Health Survey Data.

**Method:**

Data on 868 sexually active men residing in the Gambella region were extracted from the 2016 Ethiopian Demographic and health Survey. Descriptive statistics and logistic regression were respectively used to summarize descriptive data and measure the statistical associations. Adjusted odds ratio and confidence intervals were respectively used to measure statistical associations between variables and their statistical significances.

**Results:**

The current study revealed that the overall prevalence of male circumcision uptake in the Gambella region was 61.2% (95% CI: 57.96,64.44). The results of multivariable logistic regression revealed that being Muslim (AOR = 9.54, 95% CI: 6.765.13.88), being Orthodox Christian (AOR = 8.5, 95%CI: 5.00–14.45), being from Poor household (AOR = 0.11, 95%CI: 0.06, 0.22), being from medium-income household (AOR = .33, 95%CI: 0.15, 0.73), listening to radio (AOR = .29, 95%CI: .16, .54), having comprehensive HIV knowledge (AOR = .44, 95%CI: .27, .71) and ever been tested for HIV (AOR = .27, 95%CI: .16,.46) were independently associated with male circumcision uptake.

**Conclusion:**

Despite all efforts made by different stakeholders to promote the provision of male circumcision in the Gambella region, its magnitude of uptake is still unacceptably low. The federal HIV prevention and Control Office and other stakeholders working on HIV prevention and control should give due emphasis to promoting HIV-related knowledge through community-based education and through religious leaders. Integrating and streamlining HIV-related education in the academic curricula, and expanding mass media coverage should also be given due consideration by the federal government and other stakeholders. The stakeholders should also give emphasis to strengthening and empowering poor sexually active men residing in the Gambella region.

## Introduction

Male circumcision is the permanent and complete removal of the foreskin (or prepuce) that covers the glans of the penis which can be performed by several conventional or surgical methods [1]. MC is performed for various reasons like a requirement by religion, conforming to societal culture and medical purpose [1, 2]. The study from a population of 237 counties showed that approximately half of the global circumcisions were done for a religious and cultural purpose [3].

The results of two RCT conducted in South Africa and Uganda equally depicted that MC reduces the risk of acquiring HIV by 60% [4, 5]. The same study from South Africa concluded that MC provides a degree of protection against acquiring HIV infection which is equivalent to what a vaccine of high efficacy would have achieved [4]. A systematic review conducted by Jonathan et al. on 60 published articles showed that MC protects HIV, cervical cancer, cervical dysplasia, herpes simplex virus type 2, chlamydia, syphilis and human papillomavirus [6]. A retrospective cohort study done in Guinea-Bissau also showed that MC reduces the risk of acquiring HIV [7].

The prevalence of male circumcision varies globally. The study done by brian et al. based on data from 237 countries estimated that the global prevalence of MC ranges from 37 to 39% [3]. According to the 2018 WHO and John Hopkins Program for International Education in Gynecology and Obstetrics (Jhpiego) estimate, More than fourteen million adolescent and adult males in east and southern Africa had undergone MC for HIV prevention in the past decade [1]. The secondary data analysis of the 2016 Ethiopian demographic and health survey (2016 EDHS) showed that the overall national prevalence of MC among males aged 15–49 years was 91% [8].

According to the 2016 WHO report, an estimated number of 710,000 people were living with HIV and there were 30,000 new infections [9]. The secondary data analysis of three successive EDHS showed the overall prevalence of HIV in Ethiopia was 1.4, 1.5 and 0.9% in 2005, 2011 and 2016 respectively. The same report depicted that the Gambella region of Ethiopia remained the highest HIV prevalence area during all the three successive surveys with the prevalence rate of 6, 6.5 and 4.8% in 2005, 2011 and 2016 respectively which were far above the national averages [10].

The Federal Democratic Republic of Ethiopia has committed to reducing new adult HIV infections by half by 2020 and to ending AIDS as a public health threat by 2030. To realize this goal, the EFDR had set six pillars of HIV prevention among which provision of voluntary medical MC (VMMC) in areas of the country where there are high levels of HIV prevalence and low levels of MC stands forth [11]. The Gambella region of Ethiopia is characterized by the highest HIV prevalence where the magnitude of MC and factors associated with it is not well studied. Therefore, the current study is aimed to study the magnitude of MC and its predictors among sexually active men aged 15–59 years using the 2016 EDHS.

## Methods

### Population

All sexually active men aged 15–59 years living in the Gambella region of Ethiopia, based on the 2016 EDHS were included in the study.

### Data source

The current study used the data of men aged 15–59 years living in the Gambella region extracted from the nationally representative 2016 Ethiopian Demographic and Health Survey. The survey was designed to provide population and health indicator estimates at the national and regional levels. The Ethiopian DHS applied probability sampling to provide nationally representative samples of men aged 15–59 years. The survey was conducted by the Ethiopian central statistical agency (CSA) and ICF International. Totally, 18,008 households were selected for the study out of which only 94.8% (17, 067) households were occupied during the survey. The interview was completed for 98% (16,650) of the occupied households. In those interviewed households, 14,795 men were identified and 12,688 men completed the interview making a response rate of 86%. The total number of eligible Men interviewed in the Gambela region was 868. Therefore, data for the current study were extracted from an individual record of 868 reproductive-age men in the Gambella region [8].

### Statistical analysis

Data analysis was carried out using Statistical package for social science (SPSS). Descriptive statistics were used to summarize descriptive data. Multivariable logistic regression was used to identify independent predictors of MC and to control confounders among sexually active men living in the Gambella region of Ethiopia. Adjusted odds ratio and confidence interval (CI) were respectively used to measure statistical associations and their statistical significances. The confidence interval was used to declare statistical significances in the final model.

### Measurements

The dependent variable, male circumcision along with socio-demographic variables (age, residence, marital status, educational status, religion, occupation, employment), frequency of listening to radio, frequency of watching television, and ever HIV test uptake were measured by directly asking respective questions to the respondents. HIV knowledge and wealth index were measured by scoring and grouping different indicator questions asked to the respondents.

### Operational definition

**Male circumcision**: in the current study a man was asked about his circumcision status (the complete removal of the foreskin from a penis) and was considered as circumcised if he answered ‘Yes’.

**Comprehensive HIV knowledge**: Each person was asked five ‘Yes/No’ type questions (Can people reduce their chance of getting HIV by using a condom every time they have sex?, Can people reduce their chance of getting HIV by having just one uninfected sex partner who has no other sex partners?, Can people get HIV from mosquito bites?, Can people get HIV by sharing food with a person who had AIDS?, and Can a healthy-looking person have HIV?) and the right answer was always labeled ‘1’. We counted the number of right answers for each person which ranged from zero to five with a mean value of 4.6. Then, a person is said to have comprehensive HIV knowledge if he scored more than the calculated mean.

## Results

### Socio-demographic characteristics, media access and HIV related factors among sexually active men aged 15–59 years in the Gambella region, 2016

A total of 868 men aged 15–59 years from the Gambella region of Ethiopia participated in the study. The mean age (+ SD) of study participants was 29.49 + 10.8 year. Nearly two-third, 587 (67.6%) of study participants were rural residents. More than half, 502 (57.8%) had been ever married and 385 (44.4%) had attended primary education. Protestant Christians were the dominant religion group (45.3%) followed by Orthodox Christian (35.4%). Regarding the employment status of the study participants, 332 (38.2%) work as an employee all year round and 357 (41.1%) were seasonal employees (Table 1). Concerning the socio-economic status of study participants, 422 (48.6%) were rich, whereas, 363 (41.8%) of them were poor. From the total participants (100%), 275 (31.7%) and 279 (32.1%) men listen to radio and watch television at least once per week respectively. Regarding comprehensive HIV knowledge and HIV test, 575 (66.2%) and 482 (55.5%) men had comprehensive HIV knowledge and ever been tested for HIV respectively (Table 1).

**Table 1:** Socio-demographic Characteristics, socio-economic characteristics, media access and HIV related factors among sexually active men aged 15–59 years in Gamblella region, 2016

Characteristics	Circumcision status		Frequency (%)
	Circumcised	Not circumcised	
**Age**			
45+	59	39	98 (11.3)
35–44	103	73	176 (20.3)
25–34	171	85	256 (29.5)
15–24	198	140	338 (38.9)
**Residence**			
Rural	305	282	587 (67.6)
Urban	226	55	281 (32.4)
**Marital status**			
Never married	230	136	366 (42.2)
Ever married	301	201	502 (57.8)
**Educational status**			
No education	68	64	132 (15.2)
Primary	255	130	385 (44.4)
Secondary	91	83	174 (20.0)
Higher	117	60	177 (20.4)
**Religion**			
Traditional	7	31	38 (4.4)
Catholic	10	33	43 (5)
Muslim	85	2	87 (10)
Orthodox	273	34	307 (35.4)
Protestant	156	237	393 (45.3)
**Occupation**			
Non professional	171	107	278 (32)
Professional	360	230	590 (68)
**Employment**			
No working	48	70	118 (13.6)
Occasional	53	8	61 (7)
Seasonal	205	152	357 (41.1)
All year	225	107	332 (38.2)
**Wealth index**			
Poor	99	264	363 (42.8)
Medium	58	25	83 (9.6)
Rich	374	48	422 (48.6)
**Frequency of listening to radio**			
Not at all	145	219	364 (41.9)
Less than once a week	159	70	229 (26.4)
At least once a week	227	48	275 (31.7)
**Frequency of watching TV**			
Not at all	167	220	387 (44.6)
Less than once a week	141	61	202 (23.3)
At least once a week	223	56	279 (32.1)
**Comprehensive HIV knowledge**			
not knowledgeable	108	185	293 (33.8)
knowledgeable	423	152	575 (66.2)
**Ever been tested for HIV**			
No	163	223	386 (44.5)
Yes	368	114	482 (55.5)

### Prevalence of male circumcision among sexually active men aged 15–59 years in the Gambella region, 2016

**Fig. 1:**
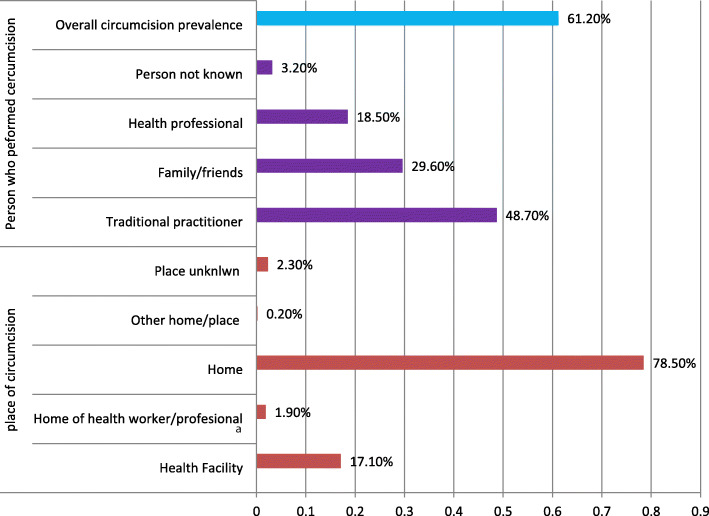
Prevalence of MC among sexually active men aged 15–59 years in Gambella region, 2016

The current study revealed that the overall prevalence of male circumcision in the Gambella region was 61.2% (95%CI: 57.96, 64.44). The study also evidenced that, more than three-fourth (78.5%) of the circumcision were performed at home, whereas only 17.1% of the circumcision were performed at health facility. Less than one-fifth of the circumcision (18.5%) were performed by health professionals, whereas 48.7 and 29.60% of the circumcision were respectively performed by traditional practitioners and family/friends (Fig. 1).

### Results of multivariable logistic regression among sexually active men aged 15–59 years in the Gambella region, 2016

Multivariable binary logistic regression was fitted to identify independent predictors of MC among men aged 15–59 years in the Gambella region. Accordingly; religion, wealth index, frequency of listening radio, comprehensive HIV knowledge and ever been tested for HIV were independent predictors of male circumcision. Muslims and Orthodox Christians were nearly 9.5 (AOR = 9.54, 95%CI: 6.77.13.88) and 8.5 (AOR = 8.50, 95%CI: 5.00–14.45) times more likely to be circumcised as compared to protestant Christians respectively. Wealth index was also an independent predictor of men circumcision among the men. Poor and medium-income men were 88.9% (AOR = 0.11, 95%CI: 0.06, 0.22) and 67% (AOR = .33, 95%CI: 0.15, 0.73) times less likely to be circumcised as compared to rich men respectively. A person who does not listen to radio was 71% (AOR = .29, 95%CI: .16, .54) times less likely to be circumcised as compared to a person who listens to radio at least once per week. A person who does not have a comprehensive HIV knowledge was 56.2% (AOR = .44, 95%CI: .27, .71) times less likely to be circumcised as compared to a person who does have comprehensive HIV knowledge. A person who has not ever been tested for HIV was 72.6% (AOR = .27, 95%CI: .16,.46) times less likely to be circumcised as compared to a person who has ever been tested for HIV (Table 2).

**Table 2:** Results of multivariable logistic regression among sexually active men aged 15–59 years in Gambella region, 2016

Variables	MC		COR 95%CI	Frequency (%)	AOR (95%CI)
	Yes	NO			
**Religion**					
Traditional	7	31	.34 (.15,.80)	38 (4.4)	.78(.27,2.29)
Catholic	10	33	.46(.22,.96)	43 (5)	.95(.46,2.50)
Muslim	85	2	64.57 (15.66,266.22)	87 (10)	9.54 (6.77,13.88) *
Orthodox	273	34	12.20 (8.10,18.38)	307 (35.4)	8.50 (5.00,14.45) *
Protestant	156	237	1	393 (45.3)	1
**Wealth index**					
Poor	99	264	.05(.03,.07)	363 (42.8)	.11(.06,.22) *
Medium	58	25	.30(.17,.52)	83 (9.6)	.33(.15,.73) *
Rich	374	48	1	422 (48.6)	1
**Frequency of listening to radio**					
Not at all	145	219	.14(.10,.20)	364 (41.9)	.29(.156,.539) *
Less than once a week	159	70	.48(.32,.73)	229 (26.4)	.60(.32,1.10)
At least once a week	227	48	1	275 (31.7)	1
**Comprehensive HIV knowledge**					
not knowledgeable	108	185	.21(.16,.28)	293 (33.8)	.44(.27,.71) *
knowledgeable	423	152	1	575 (66.2)	1
**Ever been tested for HIV**					
No	163	223	.23(.17,.30)	386 (44.5)	.27(.16,.46)*
Yes	368	114	1	482 (55.5)	1

1: reference

* statistically significant

## Discussion

In the current study the overall prevalence of circumcision among sexually active men aged 15–59 years in Gambella region was 61.2% which was by far less than the national prevalence which was 91% [8]. But the current prevalence is higher than the estimated global circumcision prevalence which ranges from 37 to 39% [3], the prevalence from South Africa which was 24% [12], the prevalence from Botswana which was 47.9% [13], the prevalence from Uganda which was 28% [14] and the prevalence from Jamaica which was only 14% [15]. The study also evidenced that, less than one-fifth of the circumcision (18.5%) were performed by health professionals, whereas, 48.7 and 29.60% of the circumcision were respectively performed by traditional practitioners and family or friends. This finding is supported by the 2016 EDHS national report which showed that 17 and 71% of the Ethiopian men were respectively circumcised by health professionals and traditional practitioners or family or friends [8].

Religion was one of the independent predictors of MC in the current study. Muslims and Orthodox Christians were nearly 9.5 (AOR = 9.54, 95%CI: 6.765.13.88) and 8.5 (AOR = 8.50, 95%CI: 5.00–14.45) times more likely to be circumcised as compared to protestant Christians respectively. This finding is consistent with other studies where Muslims and Christians were more likely to be circumcised [3, 13, 16]. This could be attributed to differences in religious requirements for circumcision by different religions [17].

Wealth index was also an independent predictor of men circumcision among sexually active men aged 15–59 years in the Gambella region. Poor and medium-income men were 88.9% (AOR = 0.11, 95%CI: 0.06, 0.22) and 67% (AOR = .33, 95%CI: 0.15, 0.73) times less likely to be circumcised as compared to rich men respectively. This finding is concordant with the result from the USA community hospitals where circumcision rates were higher in the top income quartiles [18] and finding from Ghana where highest income families received free circumcision more likely than families from lowest socio-economic quintile [19]. This may be due to the reason that people with better income may have better access to media and have a better awareness of circumcision. It could also be explained by better circumcision fee affording capacity of people with better income than those with low income.

Listing a radio was significantly associated with MC among sexually active men aged 15–59 years in the Gambella region. A person who does not listen to radio was 71% (AOR = .29, 95%CI: .16, .54) times less likely to be circumcised as compared to a person who listens to radio at least once per week. This is consistent with the result of the mixed-method study done in Zimbabwe on barriers and motivators to MC uptake where 71% of the study participants heard about MC through listening to a radio [20]. This is maybe due to the reason that males who frequently listen to radio may have a higher chance of hearing about the advantages of MC and have a high probability of being circumcised.

In the current study, comprehensive HIV knowledge was a significant predictor of male circumcision. A person who does not have a comprehensive HIV knowledge was 56.2% (AOR = .44, 95%CI: .27, .71) times less likely to be circumcised as compared to a person who does have comprehensive HIV knowledge. Although not addressed comprehensive HIV knowledge, different studies showed that being circumcised was positively associated with intention of protecting oneself from HIV acquisition [13, 20, 21].

HIV test uptake was also independently associated with men circumcision among sexually active men aged 15–59 years in the Gambella region. A person who has not ever been tested for HIV was 72.6% (AOR = .27, 95%CI: .16, .46) times less likely to be circumcised as compared to a person who has ever been tested for HIV. This finding is consistent with the results of secondary data analysis of Demographic and Health Survey from Uganda and the study from South Africa where HIV serostatus knowledge was significantly associated with male circumcision [12, 14]. This may be due to the counseling the participants do receive during HIV testing. People who receive HIV testing are often counseled on factors that expose an individual to HIV and, therefore, may have a better awareness of circumcision.

### Strength of the study

As the sampling techniques, the data collection process, and the data processing and management of the 2016 EDHS were very strong and to the standard, the results yielded from the current study are valid and dependable. Besides, the study has sufficient power as the data were extracted from large sample size.

### Limitation of the study

The temporality between male circumcision uptake and the exposure variables included in the study cannot be ascertained as the current study used data from a single cross-sectional survey, and the yielded evidence should be utilized with cautions. On the other hand, the authors failed to explore the association between socio-cultural factors and male circumcision uptake among the sexually active men in the Gambella region since qualitative data is lacking from the 2016 EDH.

## Conclusions

Even though the Ethiopian government put the provision of voluntary medical male circumcision at the heart of HIV prevention pillars in the areas where the disease is prevalent and where the level of MC is low, the magnitude of male circumcision uptake in the Gambella region of the country, where the disease is most prevalent is still unacceptably low. Religion, wealth index, frequency of listening to radio, having comprehensive HIV knowledge and ever been tested for HIV were independently associated with MC uptake. The Federal Democratic Republic of Ethiopia has committed to ending AIDS as a public health threat by 2030. However, the current low level of MC uptake among the sexually active men residing in the Gambella region of the country might be a prominent hinderance against this ambitious plan. Hence, the federal HIV prevention and Control Office (FHAPCO) and other stakeholders working on HIV prevention and control should give due emphasis to promoting HIV-related knowledge through community-based education and by using religious leaders. Integrating and streamlining HIV-related education in the academic curricula, and expanding mass media coverage should also be given due consideration by the federal government and other stakeholders. The stakeholders should also give emphasis to strengthening and empowering poor sexually active men residing in the Gambella region. Future researchers interested to the area should also address the effect of qualitative variables like socio-cultural factors on the uptake of male circumcision.

## Data Availability

All the data used in the current study are available on and openly accessed from a public domain MEASUREDHS website and its accessing link is displayed below. https://dhsprogram.com/data/dataset_admin/login_main.cfm?CFID=10106966&CFTOKEN=a531226989613ac0-7B7AD8A7-E45D-2B2E-C20F5CFFAB6B0B60

## Abbreviations

AOR: Adjusted odds ratio
CI: Confidence interval
COR: Crude odds ratio
CSA: Central statistical agency
EDHS: Ethiopia demographic and health survey
FMoH: Federal ministry of health
JHPIEGO: John Hopkins program for international education in gynecology and obstetrics
MC: Male circumcision
SPSS: Statistical package for social science
